# Age-associated hydroxymethylation in human bone-marrow mesenchymal stem cells

**DOI:** 10.1186/s12967-016-0966-x

**Published:** 2016-07-08

**Authors:** Estela G. Toraño, Gustavo F. Bayón, Álvaro del Real, Marta I. Sierra, María G. García, Antonella Carella, Thalia Belmonte, Rocío G. Urdinguio, Isabel Cubillo, Javier García-Castro, Jesús Delgado-Calle, Flor M. Pérez-Campo, José A. Riancho, Mario F. Fraga, Agustín F. Fernández

**Affiliations:** Cancer Epigenetics Laboratory, Institute of Oncology of Asturias (IUOPA), HUCA, Universidad de Oviedo, Oviedo, Spain; Unidad de Biotecnología Celular, Área de Genética Humana, Instituto de Salud Carlos III, Madrid, Spain; Department of Internal Medicine, Hospital U.M. Valdecilla, University of Cantabria, IDIVAL, Santander, Spain; Nanomaterials and Nanotechnology Research Center (CINN-CSIC)-Universidad de Oviedo-Principado de Asturias, El Entrego, Spain

**Keywords:** 5hmC, MSCs, Aging, Epigenetics, Bone-marrow

## Abstract

**Background:**

Age-associated changes in genomic DNA methylation have been primarily attributed to 5-methylcytosine (5mC). However, the recent discovery of 5-hydroxymethylcytosine (5hmC) suggests that this epigenetic mark might also play a role in the process.

**Methods:**

Here, we analyzed the genome-wide profile of 5hmc in mesenchymal stem cells (MSCs) obtained from bone-marrow donors, aged 2–89 years.

**Results:**

We identified 10,685 frequently hydroxymethylated CpG sites in MSCs that were, as in other cell types, significantly associated with low density CpG regions, introns, the histone posttranslational modification H3k4me1 and enhancers. Study of the age-associated changes to 5hmC identified 785 hyper- and 846 hypo-hydroxymethylated CpG sites in the MSCs obtained from older individuals.

**Conclusions:**

DNA hyper-hydroxymethylation in the advanced-age group was associated with loss of 5mC, which suggests that, at specific CpG sites, this epigenetic modification might play a role in DNA methylation changes during lifetime. Since bone-marrow MSCs have many clinical applications, and the fact that the epigenomic alterations in this cell type associated with aging identified in this study could have associated functional effects, the age of donors should be taken into account in clinical settings.

**Electronic supplementary material:**

The online version of this article (doi:10.1186/s12967-016-0966-x) contains supplementary material, which is available to authorized users.

## Background

Epigenetic mechanisms such as DNA methylation are implicated in many different biological processes, such as regulation of chromatin structure, X chromosome inactivation, gene imprinting, and genomic instability [1–3]. It is well known that genomic DNA methylation is modified during the lifetime of higher organisms [4], and this variation has been regularly ascribed to the epigenetic mark 5-methylcytosine (5mC) [5–8]. However, the recent discovery of the presence of 5-hydroxymethylcytosine (5hmC) in DNA implies that this epigenetic mark should also be taken into account in studies of DNA methylation and aging.

5hmC is an epigenetic modification originated through the oxidation of 5-methylcytosine by the Ten-eleven Translocation (TET) family of proteins [9, 10]. It was identified for the first time in mammals 40 years ago [11], and recent studies have shown high levels of this chemical modification in mouse Purkinje and granule neurons [12]. TET proteins also catalyze the conversion of 5hmC into 5-formylcytosine (5fC) and 5-carboxylcytosine (5caC) through consecutive oxidations [13], both of which are substrates for thymine-DNAglycosylase (Tdg), finally yielding unmethylated cytosine [14]. While the low affinity of the maintenance methyltransferase DNA methyltransferase 1 (DNMT1) for 5hmC during cell division [15] might suggest that 5hmC is merely a transient DNA modification involved in DNA demethylation pathways [15–17], the tissue-specific distribution of this epigenetic mark [18, 19] together with its high levels in the brain and central nervous system [12] point to this epigenetic modification having its own biological role. While hydroxymethylation of 5mC has been linked to the processes of demethylation during biological processes, such as primordial germ cells and zygotic development [20], several studies have associated 5hmC with transcriptional activation mediated by enhancers, both in embryonic and differentiated cells [21–25]. The functional role of 5hmC takes on greater importance given the fact that it is deregulated in various human pathologies such as cancer, and degenerative diseases like Parkinson’s and Alhzeimer’s [26, 27].

Many approaches commonly used to measure 5mC, such as conventional bisulfite modification, are in fact not able to distinguish between 5mC and 5hmC [28]. However, the development of new approaches, such as those based on the oxidation of genomic DNA with potassium perruthenate, a compound able to selectively oxidize 5hmC to 5fC, which is then converted to uracile by sodium bisulfite [29], have been a breakthrough in the study of the biological functions of 5hmC. This new possibility for analyzing the distribution of 5hmC and changes in its levels during different cellular processes will help to elucidate its biological role. For instance, recent genome-wide 5hmC studies have revealed that it may be enriched in distinct genomic regions, such as gene bodies, promoters, and distal regulatory regions in differentiated and embryonic stem cells [22, 24, 30].

Previous work from our laboratory has described a global loss of DNA methylation in bone-marrow mesenchymal stem cells (MSCs) during aging [5]. In a continuation of this research, our current contribution aims to characterize the genome wide DNA hydroxymethylation status of MSCs, obtained from bone-marrow donors aged from 2 to 89 years, using the above mentioned oxidation-based technique [29] followed by HumanMethylation 450K BeadChip arrays (Illumina®). On the one hand, our study provides information about the genomic location of 5hmC in adult stem cells, which has not been described previously, and on the other, we analyze changes in 5hmC in MSCs during aging, and their possible relationship with 5mC changes. Since bone-marrow MSCs can be used in many clinical therapies [31], the molecular characterization of these type of cells could facilitate the development of safe therapies for human diseases.

## Methods

### Isolation and culture of MSCs

MSCs were obtained from young (n: 11) and older (n: 6) bone-marrow donors. After acquiring informed consent, bone-marrow aspirates were obtained from the young patients and, from a second group, bone scrapings were obtained following hip replacement surgery. Mononuclear cells were isolated by Ficoll density centrifugation (400*g*, 25 min, 20 °C), washed twice by sedimentation with phosphate buffer (300*g*, 5 min) and the cells re-suspended in MSC medium (DMEM plus 10 % FBS) and seeded into culture flasks (Nunc, Roskilde, Denmark) at 1.5 × 10^5^ cells/cm^2^ and allowed to adhere for 24 h. MSCs were then cultured (37 °C, 5 % CO_2_) in MSC medium. DNA methylation and hydroxymethylation analyses were carried out at cell passages 4–7 (Additional file 1: Table S1). The study was approved by the Ethics Committee of Clinical Research at Hospital Universitario Niño Jesús and Hospital U.M. Valdecilla, and written informed consent was obtained from patients and parents/tutors.

### Genome-wide DNA methylation analysis with high-density arrays

Microarray-based DNA methylation profiling was performed with the HumanMethylation 450 BeadChip [32]. Oxidative bisulfite (oxBS) and bisulfite-only (BS) conversion was performed using the TrueMethyl® protocol for 450K analysis (Version 1.1, CEGX) following the manufacturer’s recommended procedure. Processed DNA samples were then hybridized to the BeadChip (Illumina), following the Illumina Infinium HD Methylation Protocol. Genotyping services were provided by the Spanish “Centro Nacional de Genotipado” (CEGEN-ISCIII) (http://www.cegen.org).

### HumanMethylation450 BeadChip data preprocessing

Raw IDAT files were processed using the R/Bioconductor package minfi (version 1.12.0). The SWAN algorithm [33] was used to correct for differences in the microarray probe designs. No background correction or control probe normalization was applied. Probes where at least two samples had detection p values >0.01, and samples where at least 5500 probes had detection p values >0.01 were filtered out. M values and beta values were computed as the final step of the preprocessing procedure.

### Batch effect correction

In order to detect whether there was any batch effect associated with technical factors, multidimensional scaling (MDS) was employed as a visualization technique to highlight any unusual interaction affecting the different samples. The posterior adjustment of the samples was performed by means of the ComBat method implemented in the R/Bioconductor sva package (version 3.12.0). The variables Sample_ID and Oxidated_Status were used as covariates of interest, while the experiment number was employed as the main batch covariate.

### Computation of hydroxymethylation levels

Beta values from oxidized samples were subtracted from their corresponding non-oxidized pair (Δß), generating an artificial dataset representing the level of 5hmC for each probe for each sample [34]. Two further datasets were created to represent 5mC and total methylation levels, using beta values from oxidized and normal samples, respectively.

### Detection of differentially methylated and hydroxymethylated probes

For the three main datasets, samples were split into two groups: young (ages ranging from 2 to 29; n: 11) and old (aged between 63 and 89; n: 6) individuals. For each of the probes in every dataset, a Wilcoxon non-parametric test was used to detect any significant change in methylation and hydroxymethylation with respect to age group. The set of p values <0.05 obtained from the tests was further adjusted for multiple comparisons using the Benjamini-Hochberg method to control for false discovery rate (FDR < 0.25) in the identification of differentially *methylated and hydroxymethylated* probes. An additional threshold of shift size was applied, filtering out the 30 % of those probes with the smallest average change between age groups.

### Identification of hydroxymethylated probes

5hmC levels were employed to generate a set of probes representing the regions where this mark was located. Probes having a hydroxymethylation level above 0.1 in at least 50 % of the samples were selected as candidates.

### Histone enrichment analysis

In order to analyze the enrichment of histone marks on a subset of probes, we used the information contained in the UCSC Genome Browser Broad Histone track from the ENCODE Project. Histone mark peaks were downloaded for every combination of cell line and antibody. For each track, a 2 × 2 contingency table was built to represent the portion of the whole set of possible probes in the microarray with respect to membership of the subset of interest, and any overlap between probes and histone peaks. A Fisher’s exact test was used to determine whether there was a significant enrichment of the selected histone mark in the subset of interest. P values were adjusted for multiple comparisons using the Benjamini-Hochberg method to control the FDR. A significance level of 0.05 was used to determine whether combinations of each histone mark and cell line showed significant changes in proportion of enrichment. Additionally, the base-2 logarithm of the odds ratio (OR) was used as a measure of shift size.

### Chromatin segment enrichment analysis

Data representing the results from the Broad ChromHMM Project were downloaded from the UCSC Genome Browser site. Each of the tracks in this dataset represents a different segmentation generated by a Hidden Markov Model (HMM) with chromatin immunoprecipitation sequencing (ChIP-Seq) signals from the Broad Histone Project as inputs. The segmentations were later curated and labeled according to their functional status [35, 36]. In order to detect significant enrichment in the proportion of probes in a given subset of interest belonging to a functional category, an analysis strategy similar to that employed for the detection of histone enrichment was performed, although in this case, a 2 × 2 contingency table was built using functional status as the basis of segmentation rather than different antibodies. Fisher’s exact test was employed to identify enrichment. Significant combinations were detected using an FDR (Benjamini-Hochberg procedure) threshold of 0.05. The base-2 logarithm of the OR was used as a measure of shift size.

### Genomic region analysis

The probes in the microarray were assigned a genomic region according to their position relative to the transcript information extracted from the R/Bioconductor package TxDb.Hsapiens.UCSC.hg19.knownGene (package version 3.0.0). A probe was said to be in a Promoter region if it was located in a region up to 2 kb upstream of the transcription start site (TSS) of any given transcript. Similarly, a set of mutually exclusive regions were defined within the transcripts, namely 5UTR, 3UTR, First Exon, Exon and Intron. A probe could belong to only one of these categories, and if the probe overlapped with two or more of those regions in different transcripts it was assigned to the region with the higher level of precedence (as dictated by the order stated above). If a probe was not assigned to any of these special regions, it was labeled by default as Intergenic. A contingency table was built for each of the subsets, partitioning the whole set of probes according to the genomic region they belonged to and the subset of interest. A Pearson’s χ^2^ test was used to determine if there was a significant change in proportion between the number of probes marked as belonging to a given region inside and outside the subset of interest. A significance level of 0.05 and the shift size, as measured by the OR, were used for this test.

### CGI status analysis

As for the genomic region analysis, probes were labeled according to their position relative to CpG-islands (CGI). The CGI locations used in the analyses were obtained from the R/Bioconductor package FDb.InfiniumMethylation.hg19 (package version 2.1.999). The procedure for generating these CGIs is described by Wu et al. [37]. “CpG shores” were defined as the 2 kbp regions flanking a CGI. “CpG shelves” were defined as the 2 kbp regions either upstream or downstream from each CpG shore. Probes not belonging to any of these regions were assigned to the special category “non-CGI”. Each probe was assigned to only one of the categories. A 4 × 2 contingency table was constructed for every subset of probes to study the association between the given subset and the different CGI categories. A χ^2^ test was used to determine if any of the categories had a significant association with the given subset. For each of the CGI status levels, a 2 × 2 contingency table was defined and another χ^2^ test was used to independently evaluate the association of the given subset with each status level, a significance level of 0.05 being used for all tests. Shift size was reported as the OR for each of the individual tests.

### Density of CpG analysis

For each of the probes in the HumanMethylation450 microarray, density of CpG was measured as the number of “CG” 2-mers present divided by the number of those possible in a 2 kbp window centered on the CpG under study. A Wilcoxon non-parametric test was used to determine if there was a significant difference between the densities of the CpGs belonging to each subset of interest and the densities of the array probes in the background. A significance level of 0.05 was employed for all tests. Shift size was measured using Cliff’s Delta (D).

### Gap distance analysis

Distance to both the centromere and telomere was measured for each of the probes in the HumanMethylation450 microarray. In order to find significant differences between the probes within the subset of interest and those in the background, a Wilcoxon non-parametric test was used. Again, a significance level of 0.05 was employed for all tests, and Cliff’s Delta (D) was used as a measure of shift size.

### Microarray background correction

Although it is sometimes referred to as a genome-wide solution, the HumanMethylation450 BeadChip only covers a fraction of the entire genome. In its 27K predecessor, the probes were mainly located at gene promoter regions, while in addition to the promoter probes, the HumanMethylation450 BeadChip includes probes located inside genes and in intergenic regions [38].

The irregular distribution of probes can lead to unwanted bias when studying whether a selected subset of probes is enriched with respect to any functional or clinical mark. A reference to the background distribution of features was included in every type of statistical test performed in order to prevent our conclusions being driven by the irregular distribution of probes. In qualitative tests (CGI status, genomic region, and histone mark enrichment), the contingency matrix was built to represent the background distribution of the microarray. In quantitative tests (CpG distance to centromeres and telomeres) the corresponding metric was compared between the subset of interest and the remaining probes in the microarray. Thus, any significant result would indicate a departure from the fixed background distribution and ignore any manufacturer bias [39].

### Circos data track smoothing

In order to plot the CpG information on a Circos genome-wide graphs [40], smoothing was applied to our data. Broad histone peak information from the UCSC Genome Browser was averaged by dividing the genome into intervals of 100 kbp and assigning each with score corresponding to the average of the broad peak scores found within it. CpG locations were enlarged to 200 kbp regions centered on the CpG in order to enhance the visualization.

### Gene ontology analysis and annotation

Probe subsets were converted to gene subsets using the annotation information from the R/Bioconductor package TxDb.Hsapiens.UCSC.hg19.knownGene [Carlson M. TxDb.Hsapiens.UCSC.hg19.knownGene: Annotation package for TxDb object(s)]. A probe was assigned to a gene if it was located within one of the genomic regions represented by the different transcripts belonging to that gene, or in a 2 kbp region upstream of the corresponding TSS. Probes converted in this way can be assigned to zero (i.e. intergenic probes) or multiple genes. After gene conversion, each subset of interest was analyzed using the HOMER software tool [41] configured to use the full set of genes represented in the HumanMethylation450 architecture as a background. HOMER tested the genes in each subset of interest against 21 different databases, including the gene ontology (GO) for biological process.

### Bisulfite pyrosequencing

DNA hydroxymethylation patterns of representative CpG sites differentially 5-hydroxymethylated (d5hmC) during aging were analyzed by bisulfite pyrosequencing in 11 of the 17 MSC samples obtained from individuals of different ages (Additional file 1: Table S1). Oxidative bisulfite (oxBS) and bisulfite-only (BS) conversion was carried out according to the TrueMethyl® Array Kit User Guide (CEGX, Version 2) with some modifications. Briefly, DNA samples were cleaned using Agencourt AMPure XP (Beckman Coulter), and then oxidized with 1 μL of a KRuO4 (Alpha Aeser) solution (375 mM in 0.3 M NaOH). Finally, BS conversion was performed using Epitect bisulfite kit (Qiagen®).

The set of primers for PCR amplification and sequencing were created using the specific software PyroMark assay design (version 2.0.01.15) and designed to hybridize with CpG free sites to ensure methylation independent amplification (Additional file 2: Table S2). After PCR amplification of the region of interest with the specific primers, pyrosequencing was performed using PyroMark Q24 reagents, and vacuum prep workstation, equipment and software (Qiagen®).

### Data analysis workflow

All the necessary steps for upstream and downstream analyses were defined and implemented using the Snakemake tool [42], which allows data scientists to generate a reproducible and inherently parallel processing pipeline. The source code of the workflow is included as Additional files.

## Results

### DNA hydroxymethylation profiling in MSCs

To study the genomic distribution of 5hmC in MSCs we determined the DNA methylation status of 479,423 CpG sites in 17 independently isolated primary MSCs, obtained from individuals aged between 2 and 89 years (Additional file 1: Table S1).

We identified 134,693 hydroxymethylated CpG sites present in at least one of the 17 MSCs samples. Interestingly, although the number of hydroxymethylated CpG sites per sample ranged from 15,761 to 42,392 (data not shown), only 10,685 (Additional file 3: Table S3) of these CpG sites were hydroxymethylated in more than 50 % of the samples. The genomic distribution of these frequently hydroxymethylated CpGs sites (FhmC sites) was characterized compared to the CpG sites analyzed by the 450 k array.

**Fig. 1:**
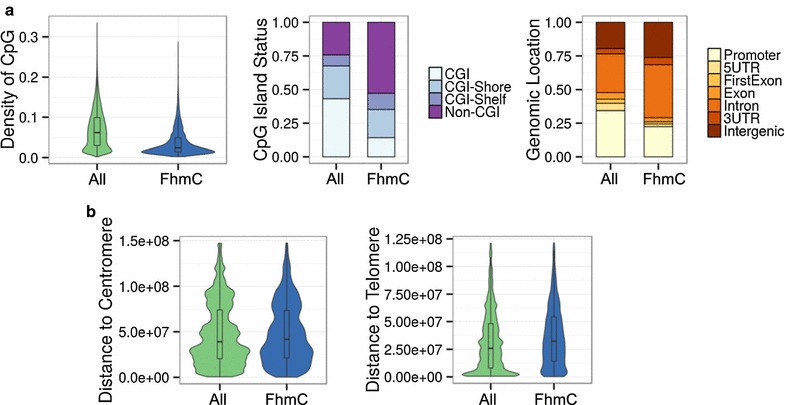
Functional genomic analysis of 5hmC. **a** *Left:* *Violin plot* showing the distribution of CpG density for the frequently hydroxymethylated sites (FhmC) and the background sites in the Infinium HumanMethylation450 microarray (Wilcoxon non-parametric test; p < 0.001, D = −0.47). *Center: Stacked bar* chart showing the relative proportions of CpG sites classified according to their CpG Island status in the frequently hydroxymethylated and background sites (Chi square test; p < 0.001, OR 3.62). *Right:* *Stacked bar* chart describing the classification of FhmC and background sites according to their relative genomic location (Chi square test; p < 0.001, OR 1.63). **b** *Violin plots* showing the distances to centromeres (*left*) and telomeres (*right*) of the FhmC sites and background probes (Wilcoxon non-parametric test; p < .01; D = 0.02 and p < .001; D = 0.06, respectively)

FhmC sites were preferentially found at the low density CpG DNA regions interrogated by the array (Wilcoxon non-parametric test; p < 0.001, D = −0.47) (Fig. 1a, left). Consequently, these FhmC sites were most frequently found in non-CpG islands (non-CGIs) (Chi square test; p < 0.001, OR 3.62) and were impoverished in CGIs (Chi square test; p < 0.001, OR 0.21) with respect to all CpG sites (Fig. 1a, center). The gene location study showed that these FhmC sites were mainly found in introns (Chi square test; p < 0.001, OR 1.63) and, at lower levels, at promoter regions (Chi square test; p < 0.001, OR 0.55), 5′UTR regions (Chi square test; p < 0.001, OR 0.35), first exon (Chi square test; p < 0.001, OR 0.53), and exons (Chi square test; p < 0.001, OR 0.58) (Fig. 1a, right). With respect to centromeres and telomeres, the FhmC sites were farther away from them than the median in terms of other background sites (Wilcoxon non-parametric test; p < 0.001 for both measurements), although the size of these shifts was rather small (D = 0.01 and D = 0.06, respectively) (Fig. 1b).

**Fig. 2:**
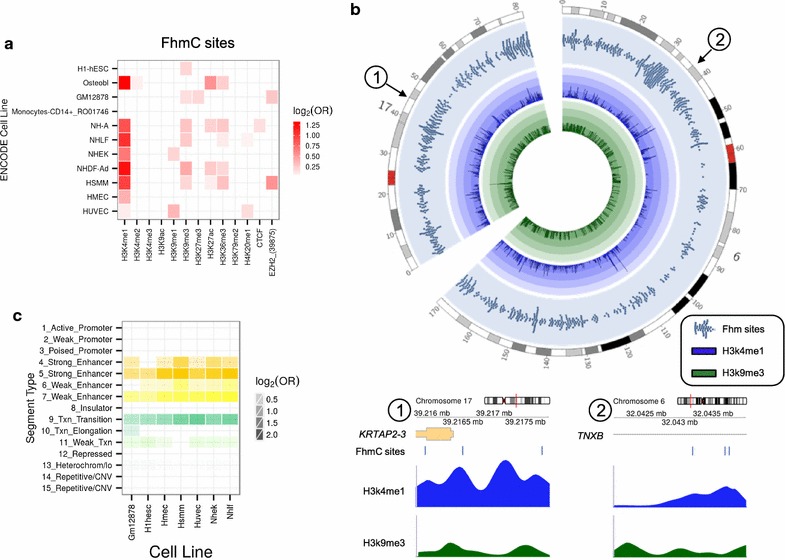
Chromatin signatures associated with DNA hydroxymethylation in MSCs. **a** *Heatmap* showing the relative enrichment of the frequently hydroxymethylated (FhmC) sites for several combinations between ENCODE Broad Histone project cell lines and histone marks. A *red tile* is drawn only if the 5hmC site is significantly enriched for the corresponding combination (Fisher’s exact test; p < 0.05); a *white* to *red* *scale* is used to represent the strength of the enrichment, using the base-2 logarithm of the odds ratio (OR) as a measure of the shift size. **b** *Upper: Circular* layout of chromosomes 6 and 17. *Outermost track*: *Tile chart* describing the location of the FhmC sites. CpG locations were enlarged to 200 kbp regions centered on the site of interest to enhance the visualization. *Middle* and *innermost tracks: Histogram charts* showing the peak scores obtained from the Broad Histone UCSC data for H3k4me1 (*middle*) and H3k9me3 (*innermost*) averaged over 200 kbp regions. *Lower*: *Genome diagrams* showing the relative enrichment of H3k4me1 and H3k9me3 marks on two specific regions of chromosomes 17 (*left*) and 6 (*right*) and the FhmC sites. **c** *Heatmap* showing the relative enrichment of FhmC sites for given sets of combinations between ENCODE Broad ChromHMM project cell lines and chromatin states. A *tile* is drawn only if FhmC sites are significantly enriched for the corresponding combination (Fisher’s exact test; p < 0.05). *Tile colors* are chosen to resemble those used in the UCSC representation of the ChromHMM dataset. The intensity of the *color* is used to represent the strength of the enrichment, using the base-2 logarithm of the OR as a measure of shift size

To identify possible chromatin marks associated with FhmC sites in adult MSCs we compared them with previously published data on a range of histone modifications and chromatin modifiers in 11 different cell types (see “Methods” section) (Fig. 2a). We found statistically significant preferential associations (Fisher’s exact test; p < 0.05) of FhmC sites with the active histone mark H3k4me1, and also with the repressive histone mark H3K9me3 (Fig. 2). A common set of “chromatin states” across seven human cell types were learned by computationally integrating ChIP-seq data for nine factors combined with input from a Hidden Markov Model (HMM) (see “Methods” section). In total, fifteen states were used for the segmentation of the genome, which were then grouped and colored to highlight predicted functional elements. We found that FhmC sites were significantly associated with enhancers (Fisher’s exact test; p < 0.05) (Fig. 2c).

### Age-associated changes in 5hmC and relationships with 5mC

**Fig. 3:**
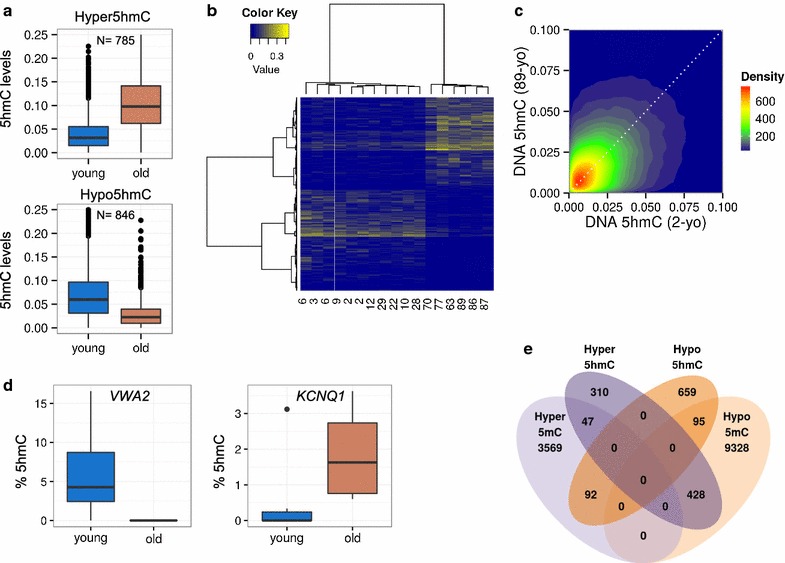
DNA hydroxymethylation changes in MSCs of two different human age groups. **a** *Box plots* showing the Δß levels of both hyperhydroxymethylated (*upper*) and hypohydroxymethylated (*lower*) CpG sites for the young and older groups sampled. **b** Unsupervised hierarchical clustering and heatmap showing the Δß values for the 785 hyper5hmC and 846 hypo5hmC from all the samples. In order to cluster both sites and samples the Manhattan distance metric and Ward clustering method were used. Whenever possible, samples are arranged by age. A *blue* to *yellow* *scale* is used to represent the Δß values, from 0 (*blue*) to a maximum value of 0.43 (*yellow*). In order to enhance the visual representation, negative Δß values were set to 0 prior to clustering. **c** Two dimensional density plot showing the relationship between Δß values of youngest *(two years old; 2*-*yo)* and oldest *(89* *years old; 89*-yo) MSCs. **d** Examples of two of the aging-specific d5hmCs, which were further validated by pyrosequencing in eleven samples. For each of the genes of interest, a *box plot* of the percentage of 5-hydroxymethylation of the CpG site of interest obtained for each sample is shown. The gene on the *left* (VWA2) shows an age-dependent hypohydroxymethylation tendency, while the gene on the *right* (KCNQ1) shows hyperhydroxymethylation with respect to age. Negative values were adjusted to 0. **e** *Venn diagram* showing the overlap of the number of differentially hyper- and hypohydroxymethylated CpG sites and differentially hyper- and hypo5methylated CpG sites

To identify possible age-dependent changes in 5hmC in MSCs we compared the levels of this epigenetic modification in two age groups, one of young individuals (ages ranging from 2 to 29; n: 11) and another of those of advanced age (63–89 years; n: 6) (Additional file 1: Table S1), using a Wilcoxon non-parametric test (see “Methods” section). We identified 1631 CpG sites differentially 5-hydroxymethylated (d5hmC) between the two groups (FDR < 0.25), of which 785 (48.13 %) gained hydroxymethylation (hyper5hmC) (Additional file 4: Table S4) and 846 (51.87 %) lost hydroxymethylation (hypo5hmC) (Additional file 5: Table S5) in the older age group (Fig. 3a). Hierarchical clustering of all samples using these d5hmC sites showed the correct classification of each individual in their corresponding age group (Fig. 3b). The direct comparison of the hydroxymethylation levels of representative samples, i.e. the youngest individual (2 years old) and the oldest (89 years old), identified thousands of differentially hydroxymethylated CpG sites, albeit showing no net gain or loss of this epigenetic modification due to chronological age (Fig. 3c).

To validate the results obtained with the methylation arrays, we selected 2 of the sequences previously identified and analyzed their methylation status by bisulfite pyrosequencing in 11 MSCs obtained from individuals aged between 2 and 87 years old (Additional file 1: Table S1). The sequences selected corresponded to the potassium voltage-gated channel subfamily Q member 1 (*KCNQ1*) gene, which become hyperhydroxymethylated with aging, and the von Willebrand factor A domain containing 2 (*VWA2*) gene, which become hypohydroxymethylated. Bisulfite pyrosequencing confirmed the hydroxymethylation changes identified with the methylation arrays (Fig. 3d).

The analysis of the gene ontology (GO) of the genes associated with d5hmC showed that both hyper- and hypomethylated genes were significantly enriched (FDR < 0.05) in specific GO terms of biological processes related to development (Additional file 6: Table S6, Additional file 7: Table S7). While both hyper- and hypohydroxymethylated genes were associated with cell adhesion, those which were hyperhydroxymethylated were also related to morphogenesis (Additional file 6: Table S6), but hypohydroxymethylated genes were more strongly associated with differentiation (Additional file 7: Table S7).

To study the relationship between age-associated changes in 5mC and 5hmC, we compared d5hmC with CpG sites differentially 5-methylated (d5mC). We first identified 13,559 CpG sites with changed 5mC levels (FDR < 0.25) of which, in the older age group, 3708 (27.34 %) gained 5-methylcytosine (hyper5mC) and 9851 (72.65 %) lost it (hypo5mC). Interestingly, most of the CpG sites hyperhydroxymethylated with aging (54.52 %) overlapped with CpG sites which lose 5-methylcytosine during aging (Fig. 3e). GO analysis of genes asociated with these common CpG sites showed significant enrichment (FDR < 0.05) in specific GO terms of biological processes related to developmental aspects, and specifically to negative regulation of the cell cycle (Additional file 8: Table S8). On the other hand, despite a small percentage of probes which lost 5hmC with age being associated with d5mC, no preferential associations between hypo5hmC and either hyper- or hypo5mC were found (Fig. 3e). To study in detail the relationships between 5mC and 5hmC we performed the same analysis again, but this time taking into account CpG island status and genomic region (Additional file 9: Figure S1). In this manner, to study the changes of 5hmC in promoters and CGI where classically 5mC have been found hypermethylated during aging, we crossed referenced CpG sites identified in both groups, but no overlap worth mentioning was found (Additional file 9: Figure S1).

### Genomic analysis of hyper5hmC and hypo5hmC CpG sites in the two age groups

To study the genomic characteristics of d5hmC in both age groups, we first determined the distribution of the hyper5hmC and hypo5hmC sites on the genome.

**Fig. 4:**
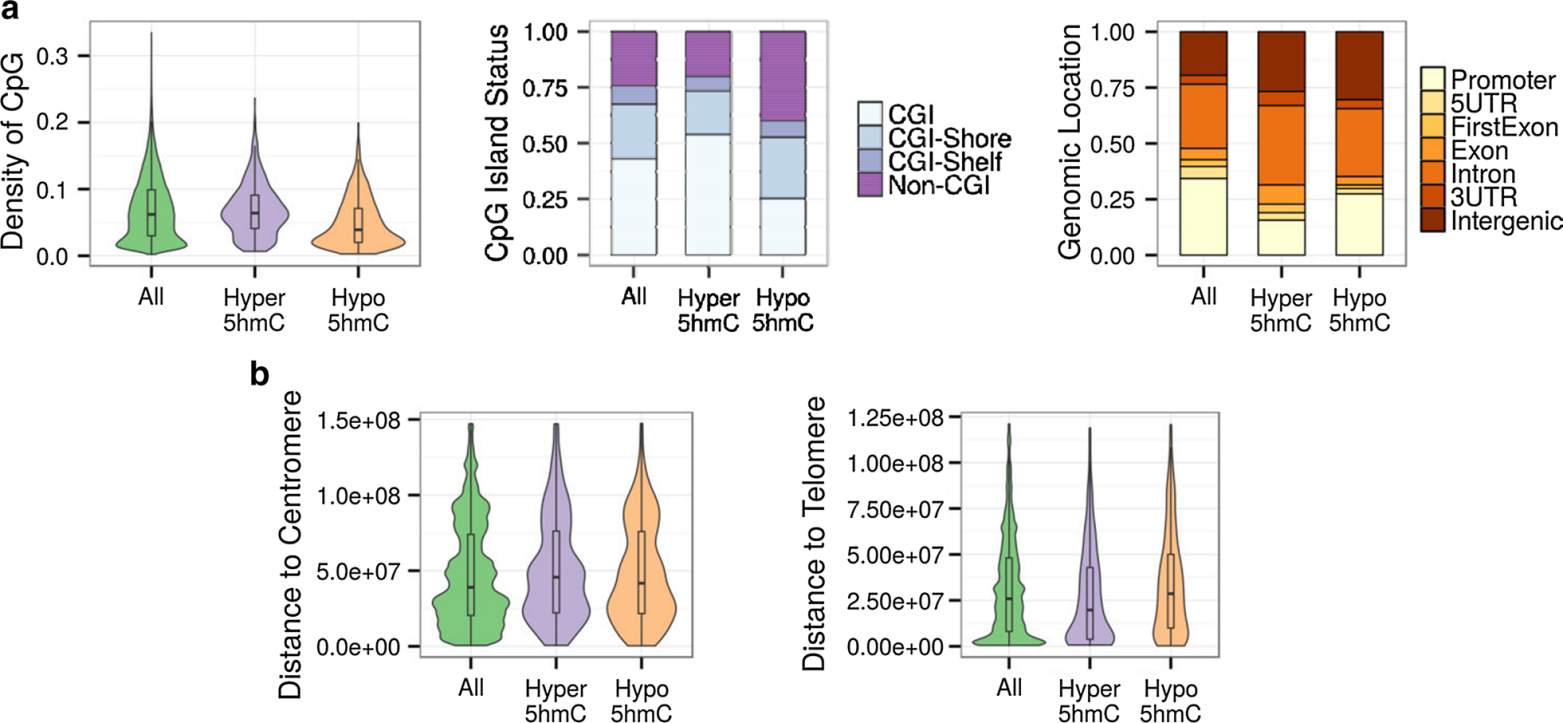
Functional genomic analysis of 5hmC changes in MSCs of two different human age groups. **a** *Left: Violin plot* showing the distribution of CpG density for hyper5hmCs and hypo5hmC sites against the background sites in the Infinium HumanMethylation450 microarray (Wilcoxon non-parametric test; p = 0.23, D = 0.02 and p < 0.001, D = −0.25, respectively). *Center: Stacked bar* chart showing the relative proportions of CpG sites, classified according to their CpG Island status, for the hyper- and hypo5hmC sites, and background probe sets. Hyper5hmCs sites are preferentially located in CpG Island regions (Chi square test; p < 0.001, OR 1.54), whereas hypo5hmC sites are preferentially located in non-CpG Island regions (Chi square test; p < 0.001, OR 2.09).* Right*: Stacked bar chart describing the classification of hyper5hmC and hypo5hmC sites and background sites according to their relative genomic location. Both are enriched in intergenic regions (Chi square test; p < 0.001, OR 1.51 and p < 0.001, OR 1.80, respectively). **b** *Violin plots* showing the distribution of distances to centromeres (*left*) (Wilcoxon non-parametric test; p = 0.006 D = 0.06, and p = 0.23 D = 0.02, respectively) and telomeres (*right*) for the hyper5hmC and hypo5hmC sites and background probe sets (Wilcoxon non-parametric test; p < 0.001 D = −0.16, and p = 0.01 D = −0.01, respectively)

The distribution of d5hmC CpG sites in the two age groups showed that hypo5hmC sites were located in regions with low CpG density (Wilcoxon non-parametric test; p < 0.001, D = −0.25) (Fig. 4a, left) and enriched in non-CGIs (Chi square test; p < 0.001, OR 2.09). Hyper5hmC sites, in contrast, were enriched in CGIs (Chi square test; p < 0.001, OR 1.54) (Fig. 4a, center). With regard to gene location, hyper5hmC sites were enriched in introns (Chi square test; p < 0.001, OR 1.36), in exons (Chi square test; p < 0.001, OR 1.78), and in 3′UTR regions (Chi square test; p < 0.001, OR 1.62). Both hyper- and hypo5hmC sites were enriched in intergenic regions (Chi square test; p < 0.001, OR 1.51 and p < 0.001, OR 1.80, respectively), and impoverished in promoter regions (Chi square test; p < 0.001, OR 0.35 and p < 0.001, OR 0.72 respectively) and 5′UTR regions (Chi square test; p < 0.001, OR 0.60 and p < 0.001, OR 0.43 respectively). Besides, hypo5hmC sites were depleted in first exons (Chi square test; p < 0.001, OR 0.53) (Fig. 4a, right). Hyper5hmC sites were found to be farther from centromeres than from other CpG sites (Wilcoxon non-parametric test; p = 0.006), although the difference was not great (D = 0.06), whereas we found no significant difference in the case of hypo5hmC (Wilcoxon non-parametric test; p = 0.23) (Fig. 4b, left). In addition, hyper5hmC sites were closer to telomeres than to other CpG sites in the array (Wilcoxon non-parametric test; p < 0.001 D = −0.15) (Fig. 4b, right).

**Fig. 5:**
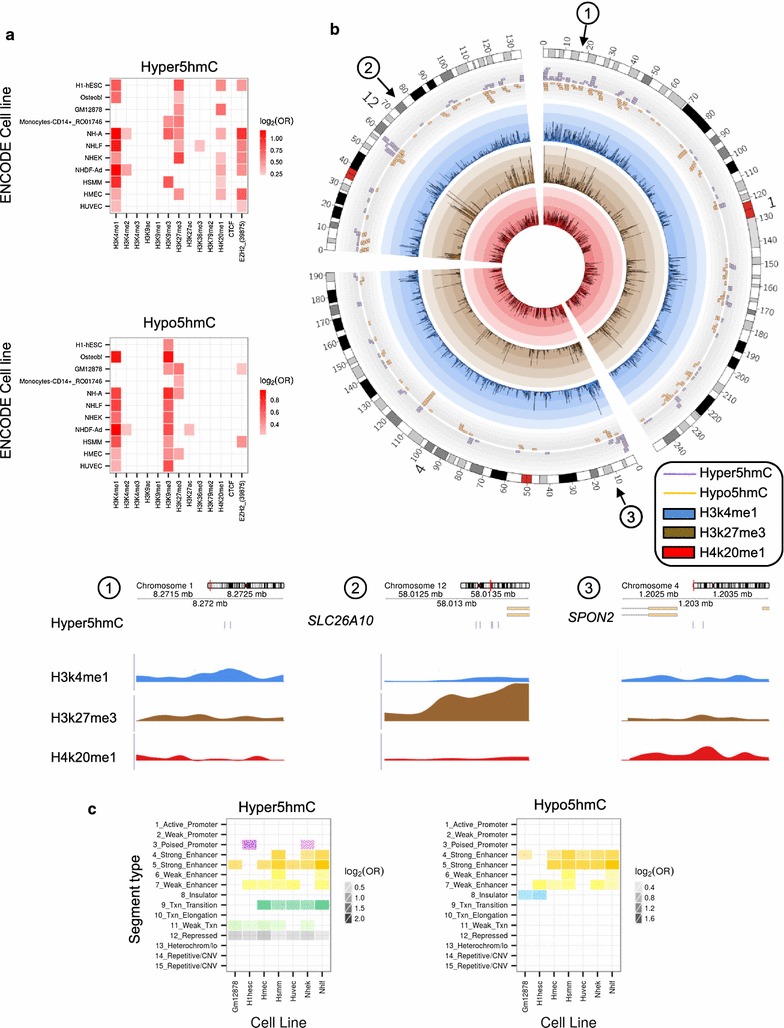
Chromatin signatures associated with DNA hydroxymethylation in MSCs of two different human age groups. **a** *Heatmap* showing the relative enrichment of the hyper5hmC (*upper*) and hypo5hmC (*lower*) sites for a given set of combinations between ENCODE Broad Histone project cell lines and histone marks. A *red tile* is drawn only if the 5hmC sites are significantly enriched for the corresponding combination (Fisher’s exact test; p < 0.05). A *white* to *red scale* is used to represent the strength of the enrichment, using the base-2 logarithm of the odds ratio (OR) as a measure of shift size. **b** *Upper: Circular layout* of chromosomes 1, 4, and 12. *Outermost track*: *Tile chart* describing the location of the hyper5hmC and hypo5hmC sites. CpG locations were enlarged to 200 kbp regions centered on the site of interest in order to enhance visualization. *Middle* and *innermost tracks*: *Histogram charts* showing the peak scores obtained from the Broad Histone UCSC data for H3k4me1 (*blue*), H3k27me3 (*brown*) and H4k20me1 (*red*) modifications on the HSMM cell line, averaged over 200 kbp regions. *Lower*: *Genome diagrams* showing the relative enrichment of H3k4me1, H3k27me3 and H4k20me1 marks on three specific regions of chromosomes 17 (*left*) and 6 (*right*) with hyper5hmCs sites. **c** *Heatmap* showing the relative enrichment of the hyper5hmCs (*left*) and hypo5hmC (*right*) sites for a given set of combinations between ENCODE Broad ChromHMM project cell lines and chromatin states. A *tile* is drawn only if the 5hmC sites are significantly enriched for the corresponding combination (Fisher’s exact test; p < 0.05). *Tile colors* are chosen to resemble those used in the UCSC representation of the ChromHMM dataset. Intensity of *color* is used to represent the strength of the enrichment, using the base-2 logarithm of the OR as a measure of shift size

Following the same methodology outlined above for the chromatin studies, we found that both hyper- and hypo5hmC CpG sites were associated with the active histone mark H3k4me1. Hypo5hmC sites were also associated with the repressive histone modification H3K9me3 (Fig. 5a, b), while hyper5hmC sites were associated with the repressive mark H3k27me3 and its methyltransferase (EZH2) and, to a lesser extent, with H4k20me1 (Fig. 5a, b). All associations were statistically significant (Fisher’s exact test; p < 0.05).

Finally, we also found statistically significant associations of both hyper- and hypo5hmC sites with enhancers (Fisher’s exact test; p < 0.05). Interestingly, hyper5hmC sites were also associated with repressed genomic regions (Fisher’s exact test; p < 0.05) and transcription-transition chromatin (Fig. 5c).

## Discussion

Previous genome-wide DNA methylation studies have shown a gradual decrease in global DNA methylation, and increased promoter-associated methylation during human aging in most cell and tissue types [6–8]. Recently, we also identified age-associated genome wide global DNA hypo-methylation changes in MSCs from humans of different ages [5].

All these studies were focused on 5mC, a stable covalent DNA modification, but the fact that it can be enzymatically converted to 5hmC provides new perspectives on the regulatory processes dependent on 5mC presence (or absence). Thus knowledge of changing DNA methylation patterns during aging (as well as under pathological conditions) needs to be complemented to take into account the contribution of the epigenetic mark 5hmC makes to these changes. Information regarding 5hmC in mammalian aging is rather limited: mouse cerebellum and hippocampus showed age-associated increased 5hmC levels [43], and increased 5hmC levels were observed in developmentally activated genes in mice brain during aging [44]. In the main these studies involved central nervous system tissues, where 5hmC is substantially enriched relative to other tissues and cell types: for instance, levels are up to tenfold greater in brain than in embryonic stem cells [12, 44]. We therefore undertook the analysis of 5hmC DNA content at the genome-wide scale, using the same MSCs previously studied [5], aiming to complete our knowledge of DNA methylation changes during human aging in this cell type. Concomitantly, we also identified thousands of frequently hydroxymethylated CpG sites in the DNA and determined their genomic distribution in MSCs.

Furthermore, the possible role of 5hmC in the epigenetic tuning of enhancer regions has been widely hypothesized [21–25, 30]. These enhancer regions can be found in two different states: active, marked by H3k4me1 and H3k27ac, and poised, marked only by H3k4me1 [45]. Our results showed an association of 5hmC CpG sites with H3k4me1, but not with H3k27ac, suggesting that, in MSCs, 5hmC is preferentially associated with poised enhancers. Although this characteristic has also been found in previous studies, with both embryonic stem [30] and differentiated cells [24], other reports have described 5hmC as being associated with active enhancers [22, 23], and linked it to developmentally important genes that can be rapidly activated or silenced [46].

Accumulation of high levels of 5hmC within gene bodies in ESCs [47] and differentiated cells [21, 44] have been previously described, and our results confirm these 5hmC enrichments in gene bodies, particularly in intronic sequences of MSCs. Studies have correlated 5hmC presence in gene bodies with increased gene expression [44, 47], alternative splicing [21], and with a depletion of 5mC at the same site [48]. In contrast to the well-established role of DNA methylation at promoter regions for transcription repression, the function of gene body methylation is far from clear: since our previous results also showed 5mC depletion on MSCs gene bodies with aging [5] it could be, as has already been suggested, that 5hmC on gene bodies is a more general epigenetic feature, whereas its presence at promoters may be a specific feature of pluripotent cell types [46]. Nevertheless, while in ESCs the presence of 5hmC in gene bodies appears to be consistently correlated with gene expression [47, 49], the evidence as to whether there is a correlation between promoter 5hmC and transcription is inconsistent between studies. Further studies are needed to ascertain whether 5hmC is correlated with gene expression in MSCs.

A limitation of our study could be its use of different methods to obtain the MSCs from the young and older groups. It can be assumed that the MSCs obtained from the bone-marrow aspirates of the young group come mainly from the perivascular niche, while those obtained from the bone scrapings of the older group are most likely from the endosteal niche. However, no potential differences between those MSCs populations have been identified to date. Although in this work the two cell populations are considered similar, we cannot rule out that some of the age-related epigenetic differences seen here are due to differences associated with the different locations.

It should be also noted that our study was performed on MSCs obtained from bone-marrow donors and extended under in vitro culture conditions. It has recently been shown that prolonged in vitro culture may affect not only 5hmc, but also 5mC [50]. That said, our samples were obtained at early passages (4–6), so methylation changes should not greatly affect our results. However, the rapid erasure of 5hmC in mouse embryonic fibroblasts (MEFs) and T cells upon cell adaptation to culture systems [51] has been described, meaning that our results could be an underrepresentation of real levels of 5hmC in MSCs in vivo. In our work, these potential losses of 5hmC in MSCs should occur in the same way in samples from the two age groups due to culture conditions being the same for both simple types. Thus we might assume that the differences in 5hmC associated with age described in this study may be a reflection of changes in this epigenetic mark in regions which remain more stable than others when passed to in vitro culture conditions. The fact that the genomic distribution of 5hmC (i.e. enrichment in low CpG density regions and enhancers, and association with the histone posttranslational modification H3K4me1) agrees with reports from other experimental settings [21, 24, 25, 44, 47, 52] does, however, appear to support our findings.

In terms of 5hmC changes between MSCs from different age groups, we found age-associated patterns, but without any clear direction of locus-specific 5hmC changes attributable to age. The study of the ontologies of genes associated with either type of change (hyper- or hypo5hmC) showed relationships with biological processes related to development, and with cell adhesion terms particularly, a very important aspect related to the maintenance of stem cell niche, and consequently with bone morphogenesis [53–55].

Of note is the fact that CpG sites gaining hydroxymethylation in the advanced age group were those which lost 5mC, in accord with previous studies in differentiated cells [24, 44]. This suggests that 5hmC could play an influential role in DNA demethylation during aging and, therefore, that its behavior with respect to DNA methylation should be taken into account along with that of 5mC. We have also found that genes associated with those probes are related to ontologies of biological processes related to development, including several terms of morphogenesis, and interestingly, with negative regulation of the cell cycle, which might underlie the loss of self-renewal and proliferation previously found in aged adult stem cells, including MSCs [55–60]. On the other hand, we have found no preferential associations between probes which lose 5hmC and 5mC changes, suggesting that the former epigenetic mark could have its own role, independent of 5mC changes.

A recent study by Yan and collaborators where “adipose-derived” MSCs from older donors were treated with the DNA methyltransferase inhibitor 5-Azacytidine evidenced increases in 5hmC which were associated with decreased 5mC during the process of DNA demethylation [60]. Furthermore, this work showed that this loss of DNA methylation occurred through the induction of TET gene expression [60]. These authors also identified a loss of 5hmC together with an increase in 5mC during aging of MSCs, which they additionally associated with a decrease in proliferation rate and a loss of bone differentiation potential. Although we did not observe any clear trend of changes in 5hmC over time, it should be taken into consideration that Yan and collaborators used immunofluorescent staining, a measure of global levels of 5hmC and 5mC (around 27 million CpGs in the genome), principally represented by repetitive sequences, whilst we performed a genome-wide analysis of “bone-marrow-derived” MSCs, and at a locus-specific level (<0.5 million CpGs), which included all the genes in the human genome. In this way we have identified possible functional roles of 5hmC changes in MSCs in the developmental process during aging. On the one hand we have found increased levels of 5hmC with age in genes related to cell adhesion and morphogenesis ontologies, decreased levels of 5hmC with age in genes related to differentiation ontologies, and increased 5hmc in combination with decreased 5mC in genes related to proliferation and development ontologies (Additional file 1: Table S1, Additional file 2: Table S2, Additional file 3: Table S3, Additional file 4: Table S4, Additional file 5: Table S5, Additional file 6: Table S6, Additional file 7: Table S7, Additional file 8: Table S8). In line with this, alterations of these development processes, such as impaired bone morphogenesis, decreased proliferation and osteoblast differentiation of MSCs derived from bone-marrow during aging have been described previously [61–63]. Interestingly, these changes in 5hmC occur in chromatin regions marked by H3K4me1, H3K27me3 and H3K29me3, which have been previously associated with the “stemness” of stem cells [64–69], and could therefore be highlighting regions which suffer other epigenetic alterations of chromatin associated with aging.

The molecular mechanisms by which these changes of 5hmC occur during aging of MSCs are yet to be determined. What is clear is that TETs have a key role in these processes. The work of Yan and collaborators [60] demonstrated increased expression levels of TET2 in aged MSCs, a fact that could be behind the 5hmC changes that we observed in our samples given that, as has been shown in other cells (i.e. embryonic stem cells), the upregulation of TET enzymes is associated with increases in 5hmC [70]. In addition, and unlike other methylcytosine oxidases, TET2 has been associated with changes of 5hmC in gene bodies [71], as is the case for the hyperhydroxymethylated CpG sites identified in our study.

Finally, other authors have also suggested that the fact of both epigenetic marks (5mC and 5hmC) co-existing in the genome and the repressive transcription effect of 5mC in promoter regions, could point to the role of 5hmC in relieving the silencing effect of 5mC by preventing binding of methyl-binding proteins [46], apart from its potential involvement in demethylation. It could also be that there are proteins involved in gene regulation able to bind 5hmC specifically, but further work is needed to clarify the role of this interesting cytosine modification in the genome.

## Conclusions

We identified more than ten thousand CpG sites frequently hydroxymethylated in adult stem cells. We showed that, as in other cell types, 5hmC is enriched in low density CpG regions, introns, the histone posttranslational modification H3k4me1 and enhancers. We identified more than 1500 CpG sites showing age-associated changes in 5hmC and, most importantly, that many of these changes occur in CpG sites that lose 5mC with aging, which suggests that 5hmC is an important epigenetic mark that should be taken into account in studies of DNA methylation and aging. In addition, and given the importance of MSCs in clinical settings, the age of donors should be taken into account since the epigenomic alterations (5mC and 5hmC) which are experienced by the epigenome of these cells may affect their potential for therapeutic use in the treatment of human diseases.

## Additional files

10.1186/s12967-016-0966-x Information on the human mesenchymal stem cells included in the study.
10.1186/s12967-016-0966-x Primers used for pyrosequencing validations.
10.1186/s12967-016-0966-x List of CpG sites frecuently hydroxymethylated in MSCs.
10.1186/s12967-016-0966-x List of CpG sites hyperhydroxymethylated in MSCs during aging.
10.1186/s12967-016-0966-x List of CpG sites hypohydroxymethylated in MSCs during aging.
10.1186/s12967-016-0966-x Gene ontology analyisis of hyper5hmC CpG sites during aging. RR: relative risk is a measure of effect size describing the change of proportions between our selected set of genes and a given term; Q value is the result from the adjustment of P values in order to control the false discovery rate (FDR) using the Benjamini-Hochberg method.
10.1186/s12967-016-0966-x Gene ontology analyisis of hypo5hmC CpG sites during aging. RR: relative risk is a measure of effect size describing the change of proportions between our selected set of genes and a given term; Q value is the result from the adjustment of P values in order to control the false discovery rate (FDR) using the Benjamini-Hochberg method.
10.1186/s12967-016-0966-x Gene ontology analyisis of both hyper5hmC and hypo5mC CpG sites during aging. RR: relative risk is a measure of effect size describing the change of proportions between our selected set of genes and a given term; Q value is the result from the adjustment of P values in order to control the false discovery rate (FDR) using the Benjamini-Hochberg method.
10.1186/s12967-016-0966-x Venn diagram showing the overlap of 5mC and 5hmC differentially hyper- and hypohydroxymethylated CpG sites at promoters (left panel), and in CGIs (right panel).

## Abbreviations

5caC: 5-carboxylcytosine
5fC: 5-formylcytosine
5hmC: 5-hydroxymethylcytosine
5mC: 5-methylcytosine
BS: bisulfite-only sample
ChIP-seq: chromatin immunoprecipitation sequencing
D: Cliff’s Delta
d5hmC: CpG sites differentially 5-hydroxymethylated
d5mC: CpG sites differentially 5-methylated
hyper5mC: CpG sites which gained 5-methylcytosine
hyper5hmC: CpG sites which gained hydroxymethylation
hypo5mC: CpG sites which lost 5-methylcytosine
hypo5hmC: CpG sites which lost hydroxymethylation
CGI: CpG-islands
DNMT1: DNA methyltransferase 1
FDR: false discovery rate
FhmC sites: frequently hydroxymethylated CpGs sites
GO: gene ontology
HMM: Hidden Markov Model
MSCs: mesenchymal stem cells
MEFs: mouse embryonic fibroblasts
MDS: multidimensional scaling
OR: odds ratio
oxBS: oxidative bisulfite sample
*KCNQ1*: potassium voltage-gated channel subfamily Q member 1
TET: ten–eleven translocation
TSS: transcription start site
*VWA2*: Von Willebrand factor A domain containing 2
