# Supplementary material for: Age-associated hydroxymethylation in human bone-marrow mesenchymal stem cells
Source: J Transl Med. 2016 Jul 8;14:207. doi: 10.1186/s12967-016-0966-x (PMC4938941; doi:10.1186/s12967-016-0966-x)
Supplement: Supplementary file 9 — 10.1186/s12967-016-0966-x Venn diagram showing the overlap of 5mC and 5hmC differentially hyper- and hypohydroxymethylated CpG sites at promoters (left panel), and in CGIs (right panel). [file 12967_2016_966_MOESM9_ESM.pptx]

## Slide 1
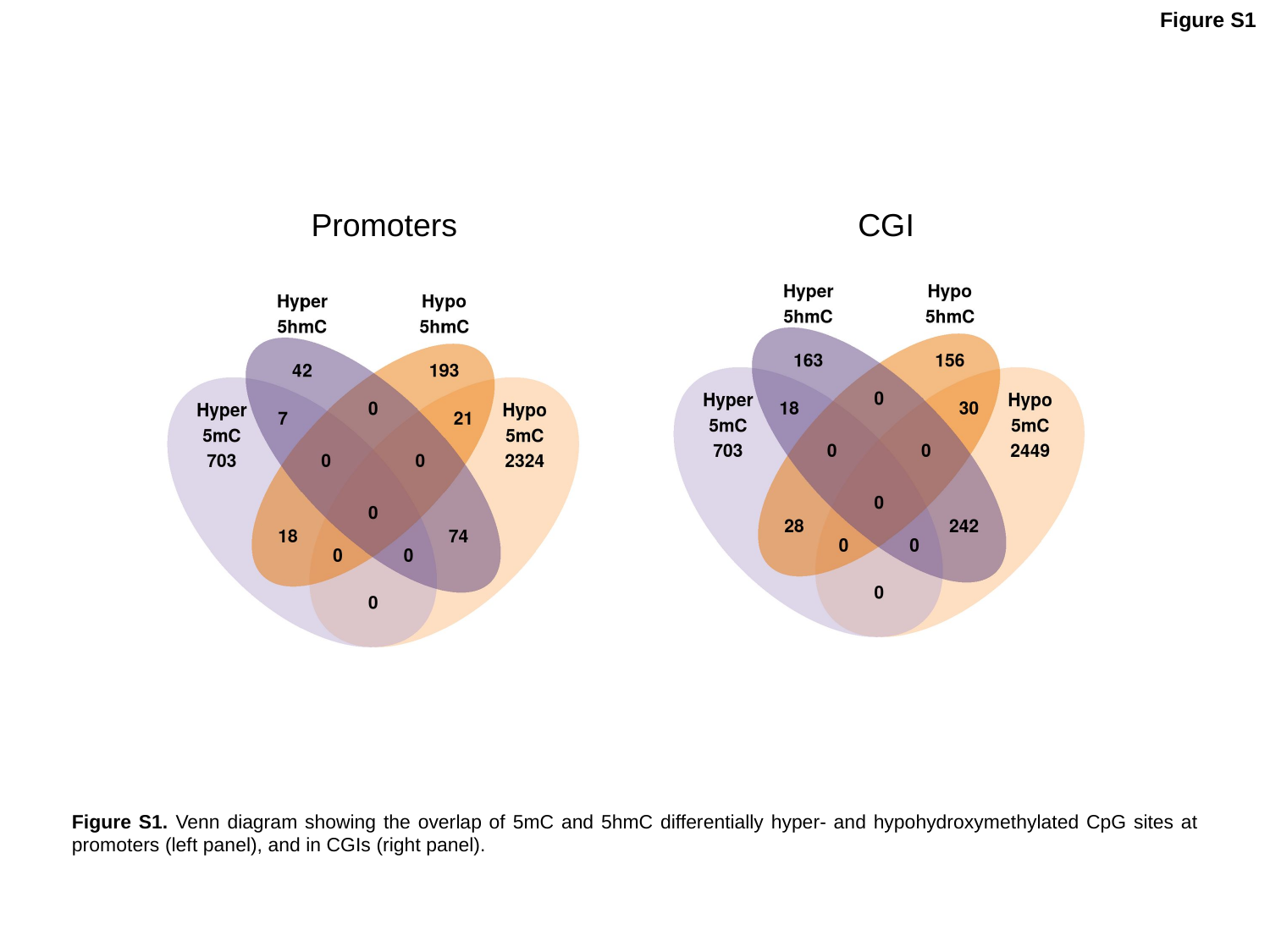

Figure S1
Promoters
CGI
Figure S1. Venn diagram showing the overlap of 5mC and 5hmC differentially hyper- and hypohydroxymethylated CpG sites at promoters (left panel), and in CGIs (right panel).
